# A Line of Sight/Non Line of Sight Recognition Method Based on the Dynamic Multi-Level Optimization of Comprehensive Features

**DOI:** 10.3390/s25020304

**Published:** 2025-01-07

**Authors:** Ziyao Ma, Zhongliang Deng, Zidu Tian, Yingjian Zhang, Jizhou Wang, Jilong Guo

**Affiliations:** School of Electronic Engineering, Beijing University of Posts and Telecommunications, Beijing 100876, China; dengzhl@bupt.edu.cn (Z.D.); 741753914@bupt.edu.cn (Z.T.); gallagher@bupt.edu.cn (Y.Z.); jizhou@bupt.edu.cn (J.W.); gjl20010323@bupt.edu.cn (J.G.)

**Keywords:** 5G CIR, LOS/NLOS recognition, time delay neural network, mamba, hierarchical features

## Abstract

With the advent of the 5G era, high-precision localization based on mobile communication networks has become a research hotspot, playing an important role in indoor emergency rescue in shopping malls, smart factory management and tracking, as well as precision marketing. However, in complex environments, non-line-of-sight (NLOS) propagation reduces the measurement accuracy of 5G signals, causing large deviations in position solving. In order to obtain high-precision position information, it is necessary to recognize the propagation state of the signal before distance measurement or angle measurement. In this paper, we propose a dynamic multi-level optimization of comprehensive features (DMOCF) network model for line-of-sight (LOS)/NLOS identification. The DMOCF model improves the expression ability of the deep model by adding a res2 module to the time delay neural network (TDNN), so that fine-grained feature information such as weak reflections or noise in the signal can be deeply understood by the model, enabling the network to realize layer-level feature processing by adding Squeeze and Excitation (SE) blocks with adaptive weight adjustment for each layer. A mamba module with position coding is added to each layer to capture the local patterns of wireless signals under complex propagation phenomena by extracting local features, enabling the model to understand the evolution of signals over time in a deeper way. In addition, this paper proposes an improved sand cat search algorithm for network parameter search, which improves search efficiency and search accuracy. Overall, this new network architecture combines the capabilities of local feature extraction, global feature preservation, and time series modeling, resulting in superior performance in the 5G channel impulse response (CIR) signal classification task, improving the accuracy of the model and accurately identifying the key characteristics of multipath signal propagation. Experimental results show that the NLOS/LOS recognition method proposed in this paper has higher accuracy than other deep learning methods.

## 1. Introduction

Eighty percent of human time is spent indoors [1]. In recent years, location services have attracted much attention, and there are various techniques for indoor localization, which are mainly classified into two categories as follows: location information acquisition relying on building infrastructure information and location information acquisition not relying on building infrastructure information [2]. Image-based indoor localization techniques and inertial sensor-based localization techniques are the typical building infrastructure-independent localization methods. Positioning methods such as Bluetooth, Wireless Fidelity (Wi-Fi), Ultra-Wideband (UWB), and mobile communication networks, measured through wireless signals, are greatly influenced by the environment and require information related to building infrastructure.

Image-based localization techniques [3,4] require high visibility of the environment, as smoke and dim environments can cause a decrease in positioning performance. Inertial sensor-based localization techniques [5,6] can obtain good localization accuracy in the initial stage of localization, but with the increase in time, the cumulative error gradually increases, resulting in unreliable localization, which needs to be corrected by high-precision wireless localization methods. The Wi-Fi-based positioning method [7,8], due to its standard Wireless Local Area Network (WLAN) design, has a signal coverage range of ten meters and does not have miniaturized nodes, resulting in a wide range of coverage that requires extremely high construction costs. In Bluetooth-based positioning methods [9,10], the propagation distance is small, the coverage is small, and Beacon node coverage is generally only about ten meters; in addition, the communication capacity is also small, resulting in an inability to broadcast fine-grained high navigation messages; the positioning accuracy is limited; and the cost of achieving wide area positioning is very high. The UWB-based positioning method [11,12] can obtain the round-trip distance between the terminal and the base station, and this method does not require strict clock synchronization between the base stations, having the characteristics of high accuracy, low complexity, and low latency; however, similar to the Bluetooth and Wi-Fi technologies, the coverage of the UWB signals is relatively small, and large-scale coverage requires an extremely high network construction cost. In addition, the existing terminals have less support for UWB, as the vast majority of terminals do not support this technology.

Wi-Fi, Bluetooth, and UWB positioning technology covers only 6% of the land and can only realize local positioning, while the 2G/3G/4G/5G network covers about 95% of the population and more than 40% of the land [13]. The use of wireless networks to achieve high-precision positioning can make up for the blind area of the satellite signal coverage in the sheltered environment, so that communication and positioning share a common network that provides seamless spatial-temporal information sensing.

With the advent of the fifth-generation mobile communication network (5G), high-precision indoor positioning based on the mobile communication network has become a research hotspot [14]. The fifth-generation New Radio (NR) network has a large bandwidth, ultra-dense networking, and large-scale antenna arrays. The increase in signal bandwidth has enabled 5G signals to gain stronger multipath resistance and smaller resolution, providing a higher ranging accuracy [15], that is, less than 6 GHz band signal bandwidth up to 100 MHz, with a millimeter wave band signal bandwidth up to 400 MHz. At the same time, the base station spacing in the 5G network can be reduced to tens of meters, which greatly guarantees the reception of high-quality signals. The large-scale antenna arrays possess the ability of angle measurement and improve the accuracy of angle measurement [16]. However, in practical applications, 5G-based localization still has some problems, among which, the NLOS problem is one of the key issues.

**Figure 1:**
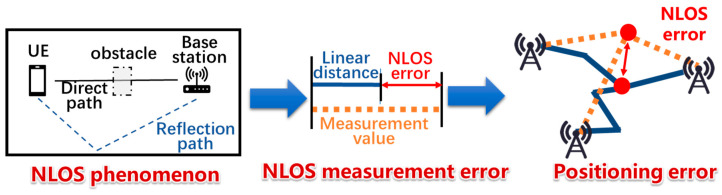
The impact of NLOS error on positioning.

NLOS is a common phenomenon in wireless signal transmission. The signal between the base station and the terminal may propagate through various paths such as diffraction, reflection, refraction, penetration, etc. [17], causing deviations in the distance and angle measurement information of 5G, finally leading to positioning deviation, as shown in Figure 1. Therefore, ways to accurately identify the NLOS are of great significance to improve the accuracy of indoor localization.

In recent years, researchers have proposed many methods for recognizing NLOS, which are mainly categorized into traditional parameter identification methods, machine learning-based methods, and deep learning-based methods. The traditional LOS/NLOS identification method utilizes the characteristic parameters of the transmission channel, such as Rice factor, kurtosis, skewness, mean excess delay (MED), etc., has low accuracy, and is only applicable to static environments. Machine learning-based methods require the manual extraction of useful information, which requires a lot of manual labor. Deep learning-based methods generally use convolutional neural networks (CNN), residual networks, SSM models, transformer models, and their combination with other techniques. The current research mainly focuses on the CNN network structure based on the spatialization of time series data and the inverse computation of temporal correlation by calculating the spatial correlation, which ignores the structurally delicate correlation of the temporal data. Although it can achieve good learning results, the efficiency is low, the redundancy is high, and it is difficult to train.

Based on this, the contributions made in this paper are as follows:

We propose the DMOCF model, which enhances the contrast of feature learning by reshaping the input CIR data structure into a distinctly characterized direct path, the reflected paths, and noise information. Res2 block and SE block were added to the TDNN. The Res2 module introduces multiple branches in each residual unit, extracting feature information at different scales through the differences in these branches, preserving rich detailed information, achieving a deep understanding of the features, and enhancing the network’s generalization ability. The SE module uses squeezing and excitation operations to obtain the importance of each feature channel, which is used as a weight to extract useful features and suppress features that are not significant for the current task. This enables the network to adaptively learn the importance of each channel and adjust the weight contribution of each channel in the features according to the task, thus achieving hierarchical feature processing. A mamba module with position coding is added to each layer to capture the local patterns of complex propagation phenomena such as the reflection, scattering, and diffraction of the input signal by extracting local features, enabling the model to understand the evolution of the signal over time in a deeper way. In addition, a residual connection design is introduced to maintain stability as the network depth increases and to prevent the gradient vanishing problem, while preserving the global properties of the inputs so that local information can be extracted without losing the overall structure of the sequence.

An improved sand cat search algorithm for network parameters is proposed to optimize the predation process of sand cat populations by introducing a nonlinear period adjustment strategy based on logarithmic function, and a dynamic adjustment method of search radius based on Quasi-opposition-based learning is proposed to enable large-scale coarse search in the initial stage and small-scale fine search in the prey attack stage, which enhances the search efficiency and search accuracy.

## 2. Related Work

In this section, related work on the recognition of LOS/NLOS is briefly presented. Firstly, the basic strategies for indoor localization in the NLOS environment are discussed; secondly, the main algorithms for LOS/NLOS classification are presented; then, the deep neural network recognition algorithms and their characteristics are discussed; finally, the challenges faced by recognition of LOS/NLOS algorithms are discussed.

### 2.1. Basic Strategies for Positioning Under NLOS Environment

Currently, many scholars have carried out research on LOS/NLOS recognition methods and have achieved significant results. According to whether the measurement statistical information can be obtained and whether the current environmental LOS situation is known or not, the localization problems in NLOS environments can be classified into the following four categories [18]:

(1) The LOS situation of the current environment is known, and measurement statistics are available [19,20]. In this case, NLOS can be suppressed to minimize its impact.

(2) The LOS situation of the current environment is known, but accurate measurement statistics cannot be obtained [21]. In this case, the impact of NLOS can be mitigated by adding weights to the measurement information or filtering the LOS base stations.

(3) The LOS situation of the current environment cannot be obtained, but accurate measurement statistics can be obtained [22,23]. In this case, the impact of NLOS can be mitigated by compensating or filtering the NLOS base stations with the measurement statistics information of the signals.

(4) It is not possible to obtain the LOS situation of the current environment and the accurate measurement statistical information. In this case, the measurement information produces a large offset, and the positioning accuracy is significantly reduced.

For the 5G system, it is more difficult to recognize the LOS situation in the current environment than to obtain accurate measurement statistics information. Therefore, recognizing the LOS/NLOS conditions of the terminal is of great practical significance for improving the positioning accuracy.

### 2.2. Traditional LOS/NLOS Recognition Methods

Recognition algorithms for LOS/NLOS are divided into three main categories, map-based recognition algorithms, recognition algorithms based on measurement information (distance measurements and angle measurements), and recognition algorithms based on channel information [24].

Map-based LOS/NLOS recognition algorithms assume that the building map is accurately known, and the map information can be utilized for the identification of the propagation channel of the signal. The ray tracing algorithm is one of the classic algorithms used for map-based LOS/NLOS recognition, which can accurately characterize the propagation state of a signal in conjunction with map information [25].

Measurement information-based LOS/NLOS recognition algorithms can judge the signal propagation state by the statistical feature distribution or residual information of these single or combined metrics, such as arrival time, arrival time difference, angle of arrival, and signal reception strength [26,27,28]. Ref. [26] decouples the multipath components to better separate between LOS and NLOS by relying on angle of arrival and arrival times.

LOS/NLOS recognition algorithms based on channel features usually use trusted channel parameters and employ threshold discrimination methods based on hypothesis testing [29], machine learning-based classification methods [30,31], and deep learning-based classification methods [32,33].

### 2.3. Deep Neural Network Based LOS/NLOS Recognition Approach

With the development of deep neural networks in recent years, many LOS/NLOS classification algorithms based on deep neural networks have been developed. The main neural network-based LOS/NLOS classification algorithms are based on CNN, Long Short-Term Memory (LSTM) architectures, and their combinations [34,35], which replace manual feature extraction and feature optimization by automatically identifying and abstracting data features through a multilayer neural network structure inside the model. They input the CIR as a time series into the neural network and realize the classification of signal propagation states after achieving a deep learning of its features.

CNN-based networks are able to preserve the spatial structure of the input data, but the fixed-size convolutional kernel of CNNs may limit their performance when dealing with variable-length sequence data, and appropriate strategies need to be adopted to deal with sequence data of different lengths. CNNs are mainly concerned with the local features of the data, and they have a weak ability for capturing global information. The shared-parameter mechanism is inflexible enough to deal with sequence data. When performing LOS/NLOS recognition, it is necessary to comprehensively consider both global and local features.

LSTM-based networks are specifically developed to solve problems related to sequential data such as language, speech, etc. CIR is also a kind of time series data, the propagation path of the signal may involve information from multiple moments. The memory units in LSTM effectively solve the problem of gradient vanishing or exploding through gating mechanisms, allowing the network to selectively remember or forget previous information and more effectively capture long-term dependencies in sequence data. However, LSTM networks are mainly used to deal with long-term dependencies and have a limited capacity to deal with purely local problems, as well as memory constraints, leading to a limited understanding of global information in the model.

### 2.4. Challenges Faced by Existing LOS/NLOS Recognition Algorithms

Existing LOS/NLOS recognition methods are diverse and can achieve considerable results, but some challenges remain.

Map-based LOS/NLOS recognition algorithms require access to a priori map information, which in many cases is not readily available and the map environment changes rapidly. Methods using ray tracing require two initial positions of the signal generator and have high computational complexity.

Distance-based LOS/NLOS classification algorithms require multiple distance measurements at the same location to establish statistical information about the variation in distance measurements and angle measurements, and they have a slow convergence rate.

Traditional machine learning-based LOS/NLOS recognition algorithms rely on manual feature extraction, as the selected features may not be able to describe the overall characteristics of the signal propagation channel, thus affecting the classification accuracy of LOS/NLOS classification.

Deep learning-based LOS/NLOS recognition algorithms do not require manual feature extraction, which improves work efficiency and accuracy, but they cannot control the relationship between global and local features well, and it is easy to lose useful information in the process of feature extraction.

### 2.5. The LOS/NLOS Recognition Scheme Proposed in This Article

This paper proposes a comprehensive feature dynamic multi-layer optimization network, which improves the feature extraction range by inflated convolution, extends the temporal attention mechanism to the channel dimension by jump connection and one-dimensional squeeze excitation (SE) block, performs hierarchical feature extraction, adds position coding, and uses a mamba module in each layer to capture the global temporal correlation on the basis of the local feature information extraction that enables the model to understand the evolution of the signal over time in more depth.

## 3. Fifth-Generation CSI-RS Signal Model and Channel Impulse Response

Fifth-generation CSI-RS signals play an important role in channel estimation due to their high efficiency in resource utilization efficiency, detailed channel state information, etc. CSI-RS is divided into two types as follows: non-zero power (NZP) CSI-RS and zero power (ZP) CSI-RS. A ZP CSI-RS does not need to be generated and mapped to the RE, and it is used for rate matching on the Physical Downlink Shared Channel (PDSCH) without broadcasting signals. A NZP CSI-RS needs to be physically generated and mapped to the resource element to be able to report the main channel status information.

The sequence generation process for 5G CSI-RS is as follows, and the reference signal sequence is given by

(1) $$r\left(m\right)=\frac{1}{\sqrt{2}}\left(1-2c\left(2m\right)\right)+j\frac{1}{\sqrt{2}}\left(1-2c\left(2m+1\right)\right)$$

where c(n) in the above equation is defined by a 31-bit gold sequence, which is generated by combining two m-sequences in the following way:

(2) $$\begin{array}{cc} c\left(n\right)= & \left(x_{1}\left(n+N_{C}\right)+x_{2}\left(n+N_{C}\right)\right)\mathrm{mod}2\\ x_{1}(n+31)= & \left(x_{1}\left(n+3\right)+x_{1}\left(n\right)\right)\mathrm{mod}2\\ x_{2}\left(n+31\right)= & \left(x_{2}\left(n+3\right)+x_{2}\left(n+2\right)+x_{2}\left(n+1\right)+x_{2}\left(n\right)\right)\mathrm{mod}2 \end{array}$$

where $N_{C}=1600$ is the truncation length and mod2 is the operation of dividing by 2 to take the remainder. For the first m-sequence, $x_{1}(n)$, $x_{1}(0)=1$, $x_{1}\left(n\right)=0,\;n=1,2,...,30$, and for the second m-sequence, $x_{2}(n)$, the initialization sequence is

(3) $$c_{\mathrm{init}}=\left(2^{10}\left(N_{\mathrm{symb}}^{\mathrm{slot}}n_{s,f}^{\mu }+l+1\right)\left(2n_{\mathrm{ID}}+1\right)+n_{\mathrm{ID}}\right)\mathrm{mod}2^{31}$$

where $n_{s,\;f}^{\mu }$ is the slot number within a radio frame, l is the OFDM symbol number within a slot, and $N_{\mathrm{symb}}^{\mathrm{slot}}$ is the OFDM symbol number within the time slot.

The CSI-RS signal is modulated by OFDM, transmitted through digital to analog conversion and carrier modulation, received through a wireless channel. Under multipath propagation conditions, the CIR of the channel is modeled as

(4) $$h\left(t,\tau \right)=\sum_{p=0}^{P-1}\beta_{p}\left(t\right)e^{-j\phi_{p}\left(t\right)}\delta \left(\tau -\tau_{p}\right)$$

where $\beta_{p}\left(t\right)$ denotes the large-scale fading coefficient, which depends on the path loss and shadow fading; $\phi_{p}\left(t\right)$ denotes the phase offset; the two stochastic processes are independent of each other; p is the number of paths; δ(·) is the Dirac function; $\tau_{p}$ is denoting the multipath delay; and $\tau_{0}=0$. It is common to let $\alpha_{p}\left(t\right)=\beta_{p}\left(t\right)e^{-j\phi_{p}\left(t\right)}$, denoting the fading coefficients of the channel. Considering t as a fixed parameter, the Fourier transform is performed on τ and the corresponding Channel Frequency Response (CFR) is modeled as

(5) $$H[t,f]=\sum_{p=0}^{P-1}\alpha_{p}\left(t\right)e^{-j2\pi f\tau_{p}}$$

Assuming that the OFDM signals at the receiver are fully synchronized, the baseband signal is sampled at $T_{s}$ intervals to obtain a discrete baseband received signal of

(6) $$y_{l}\left(kT_{s}\right)=h(kT_{s},\tau )*s_{l}\left(\tau \right)+n(kT_{s})$$

where $h(kT_{s},\tau )$ is the discrete channel impulse response, $s_{l}\left(\tau \right)$ is the discrete transmit signal, ∗ is the convolution operation, $n(kT_{s})$ is the discrete noise component, and $y_{l}\left(kT_{s}\right)$ is the discrete receive signal.

## 4. Proposed Dynamic Multi-Level Optimization of Comprehensive Features Network

### 4.1. TDNN-Based Hierarchical Feature Extraction Module

The TDNN is developed from static feed-forward neural networks for processing one-dimensional time series such as speech and text, which are essentially one-dimensional convolutions. In order to improve the sensory field of convolution, a one-dimensional expansion convolution is usually used for feature extraction. The inflated convolution can more effectively focus on the connection between the front and back temporal data, and it can even skip the temporal data of the neighboring epoch elements, connecting the nearby non-neighboring epoch elements.

**Figure 2:**
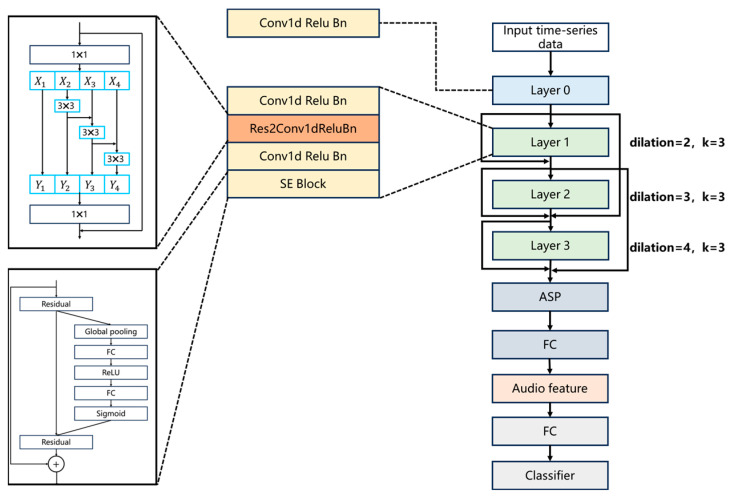
Improved TDNN incorporating res2 and SE blocks.

This article proposes some improvements to the TDNN, as shown in Figure 2. In layer 1, layer 2, and layer 3, we introduced additional skip connections and one-dimensional SE blocks to extend the temporal attention mechanism to the channel dimension, obtaining relevant information between multi-scale epoch data. In these three layers, the spacing between the convolution kernel elements is 2, 3, and 4, allowing different channels to extract information from different receptive fields.

In the process of LOS/NLOS identification, we are not only concerned about the amplitude information of the arriving signal, but also the phase information of the signal. Wireless signals are blocked by obstacles during propagation, and after reflection, diffraction, and refraction, the phase difference is generated, which leads to the 5G CIR generating peaks of delay in the timing. This is a good match with the characteristics of improved TDNN architecture, so we conduct research on LOS/NLOS identification based on TDNN architecture.

### 4.2. A Mamba Time-Varying Dynamic Tuning Module Based on Selective Space-State Modeling (SSM)

Common deep learning methods for processing time series generally use recurrent neural networks represented by RNNs and LSTMs or transformer architectures, which are capable of achieving good performance but still have some problems. Recurrent neural networks are slightly slower to train but have the ability of fast inference. The transformer architecture is able to realize parallel training, but the computational complexity of its self-attention mechanism increases by square level with the length of the data, resulting in a relatively slow inference speed. The ways of balancing the training speed and inference speed under the premise of ensuring that the performance of deep learning is not affected have become a major problem.

For this reason, the mamba model has emerged. Mamba is a selective SSM that can capture long-term dependencies in multivariate time series data. Computational complexity increases linearly with the length of the data, maintaining linear scalability and small memory usage, while also having a good learning ability.

#### 4.2.1. SSM Model

SSM models are used to describe the state representation of a sequence at each time step and to predict its next state based on inputs. The state-space model is defined as

(7) $$x^{\prime }\left(t\right)=Ax\left(t\right)+Bu(t)$$

(8) $$y\left(t\right)=Cx\left(t\right)+Du(t)$$

where u(t) is the input signal, x(t) is the hidden state, y(t) is the input signal, $A\in R^{N\times N}$ is the state evolution parameter, $B\in R^{N\times 1}$ is the input weight matrix, $C\in R^{N\times 1}$ is the output weight matrix, and $D\in R^{N}$ is the transfer function matrix. A transforms the system from the current state to the next state, representing the system’s state transition matrix and describing the dynamic characteristics within the system. B characterize the system’s input state based on the system’s overall input, and the elements in B reflect the sensitivity and response speed of the system to the input signals. C reflects the relationship between the overall state of the system and the output. D is a jump-connectivity matrix which reflects the direct influence of the system inputs on the output of the system. Since the SSM and the state transfer function can be obtained through model learning, we will omit the parameter D (or equivalently assume D=0).

**Figure 3:**
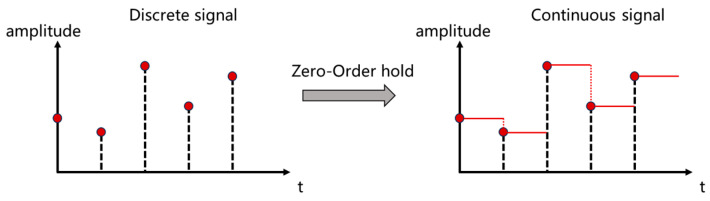
Zero order keeper diagram.

Wireless signals are usually processed using digital signals for data processing. In order to discretize the continuous function u(t), the resolution step Δ needs to be set, and the input discrete signal $u_{k}$ can be regarded as the continuous signal u(t) to be sampled, $u_{k}=u(k\Delta )$. In this paper, a zero-order keeper is used to convert the matrix A into an approximation matrix $\bar{A}$, as shown in Figure 3, and the discrete SSM is modeled as

(9) $$x_{k}=\bar{A}x_{k-1}+\bar{B}u_{k}$$

(10) $$y_{k}=\bar{C}x_{k}$$

(11) $$\bar{A}=exp(\Delta A)$$

(12) $$\bar{B}={\left(\Delta A\right)}^{-1}\left(\exp\left(\Delta A\right)-I\right)\cdot \Delta B$$

(13) $$\bar{C}=C$$

where $x_{k-1}$ and $x_{k}$ represent the hidden state of the previous epoch element and the current epoch element, respectively. It can be seen from the above equation that the current hidden state is affected by the joint influence of the hidden state of the previous moment and the input of the current epoch element. This recursive updating mechanism is similar to the basic principle of the RNN. The RNN captures time series characteristics through recursive properties, while structured state-space models accelerate the inference process by introducing recursive structures.

Bringing the previous moment state $x_{k-1}$ into the current moment state $x_{k}$ and making $x_{-1}=0$, we can obtain

(14) $$x_{t}={\bar{A}}^{2}x_{t-2}+\bar{A}\bar{B}u_{t-1}+\bar{B}u_{t}=\sum_{k=0}^{t}{\bar{A}}^{k}\bar{B}u_{t-k}$$

(15) $$y_{t}=\sum_{k=0}^{t}\bar{C}{\bar{A}}^{k}\bar{B}u_{t-k}=\bar{K}\times u$$

(16) $$\bar{K}=(\bar{C}\bar{B},\bar{C}\bar{A}\bar{B,}\ldots ,\bar{C}{\bar{A}}^{L-1}\bar{B})$$

The mathematical model of the SSM is given in the same form as the discrete convolution, with $\bar{K}$ as the convolution kernel, which means that the structured SSM can be trained in parallel using the convolutional mode and switched to the cyclic mode for efficient autoregressive inference.

#### 4.2.2. Mamba Model

**Figure 4:**
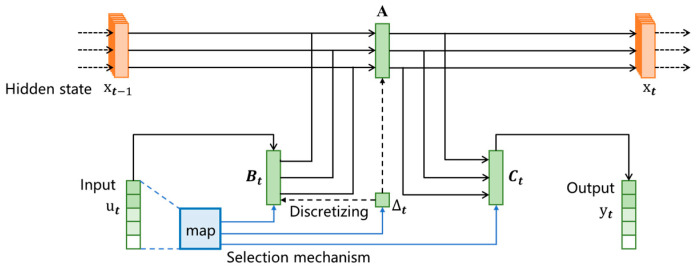
Selective SSM structure diagram.

The parameters of the equations of the structured spatial state model are constant over time and they are fixed for all time steps; they are linear time-invariant systems. Although global convolution based on linear time-invariant systems can capture temporal features using different convolution kernels, it lacks content perception and cannot clarify the focus and logic of the input. Therefore, selective state-space models have emerged, as shown in Figure 4. Make Δ change over time to $\Delta_{t}$ as the main switch for system regulation, controls the importance of the current input data. The knob that changes the mapping parameters B and C to become $B_{t}$ and $C_{t}$ as inputs and outputs is used to regulate the dynamic loop of the hidden states of input data and before and after data, transforming a linear time-invariant system into a nonlinear time varying system through a time-varying selection mechanism. The selective spatial state model can accurately control the propagation and interaction of data information in the sequence through the selective mechanism, dynamically adjust the behavior according to the sequence of input information, select and remember the key information from a large amount of information, ignore the irrelevant parts, and also maintain logic consistency when dealing with successive information, which not only improves the ability of the system to grasp the key points, but also improves reasoning when dealing with the before and after data. In addition, the selective spatial state model controls the time-varying parameters with a projection module, which realizes parameter sharing and improves the computational efficiency.

The selectivity mechanism is mainly realized by the gating mechanism

(17) $$S_{B}\left(u\right)={Linear}_{N}(u)$$

(18) $$S_{C}\left(u\right)={Linear}_{N}(u)$$

(19) $$S_{∆}\left(x\right)={Broadcast}_{D}({Linear}_{1}\left(u\right))$$

(20) $${∆}_{t}=softplus$$

where Linear is the linear layer used to construct linear transformations in neural networks, softplus is the nonlinear activation function, $S_{B}\left(u\right)$ and $S_{C}\left(u\right)$ are the input sequences that have been parameterized and projected, and $S_{∆}\left(x\right)$ and ${∆}_{t}$ are the connection elements of the gating mechanism.

### 4.3. Improved Optimization Algorithm for Sand Cat Swarms

The existing heuristic algorithms include the genetic algorithm and particle swarm optimization algorithm. The genetic algorithm simulates the law of survival of the fittest in biological evolution, while the particle swarm optimization algorithm originates from the study of bird hunting behavior. The particle swarm optimization algorithm mainly searches for the optimal solution through the cooperation between particles, emphasizing the interaction between individuals and groups. The convergence speed is slow and requires multiple iterations to achieve the search effect. Genetic algorithms search for optimal solutions through operations such as selection, crossover, and mutation, with a focus on global search and population evolution, but they may sometimes fall into local optima. The sand cat search algorithm combines the ability of global exploration and local search, focusing more on making local adjustments during the search process, which helps avoid becoming stuck in local optima and has a fast convergence speed, which can quickly find the optimal solution.

#### 4.3.1. The Mathematical Model of the Sand Cat Swarm Optimization Algorithm

The sand cat swarm optimization algorithm is a mathematical model constructed based on the foraging behavior of sand cats in the desert, which are able to locate prey above or below ground by detecting low frequency noise. The algorithm considers the optimal value in the exploration space as the prey, continuously explores the new unknown space through the position update, and finally approaches the area where the optimal value is located. The sand cat swarm optimization algorithm follows a prey search mechanism and a prey attack mechanism. The prey search mechanism simulates the process of sand cats searching for prey. The search prey equation of the sand cat population is as follows:

(21) $$x\left(t+1\right)=r\cdot (x_{b}\left(t\right)-rand(0,1)\cdot x_{c}(t))$$

where $x_{b}$ is the best candidate position for each sand cat, $x_{c}$ is the current position of the sand cat, r is the sensitivity range, and t is the number of iterations. A sand cat’s sensitivity range to noise can be described as follows:

(22) $$r_{G}=s_{M}-\frac{s_{M}\times {iter}_{c}}{{iter}_{max}}$$

(23) $$r=r_{G}\times rand(0,1)$$

where $r_{G}$ is the sensitivity range of the sand cat sensing the noise varying from 0 to 2 kHz; $s_{M}$ comes from the auditory features of the sand cat, assuming its value is 2; ${iter}_{c}$ denotes the current iteration; ${iter}_{max}$ is the maximal iteration; and rand(0,1) denotes a random number from 0 to 1.

Sand cats attack their prey at the end of a prey search, and the attack mechanism in sand cat populations is expressed as follows:

(24) $$x_{rnd}=\left|rand\left(0,1\right)\cdot x_{b}\left(t\right)-x_{c}\left(t\right)\right|$$

(25) $$x\left(t+1\right)=x_{b}\left(t\right)-r\cdot x_{rnd}\cdot cos(\theta )$$

where $x_{rnd}$ is a random position information generated from the best position and the current position; each member in the population is able to move in different circular directions move. θ is a random angle between 0 and 360, the value domain of cos⁡(θ) is [−1, 1], and the latest position of the sand cat is calculated jointly by $x_{b}\left(t\right)$ and $x_{rnd}$. The random position ensures that the sand cat is close to its prey, and the random angle prevents the algorithm from falling into a local optimum.

The sand cat swarm algorithm balances the prey search and prey attack phases by an adaptive factor R as follows:

(26) $$R=2\times r_{G}\times rand\left(0,1\right)-r_{G}$$

At different stages, the latest position of each individual sand cat can be represented as

(27) $$x\left(t+1\right)=\left\{\begin{array}{c} r\cdot \left(x_{b}\left(t\right)-rand\left(0,1\right)\cdot x_{c}\left(t\right)\right)\;\left|R\right|>1;\;search\;\mathrm{mechanism}\\ x_{b}\left(t\right)-r\cdot x_{rnd}\cdot \cos\left(\theta \right)\;\left|R\right|\leq 1;\;attack\;mechanism\;\; \end{array}\right.$$

Each sand cat has a different search radius during the exploration phase, preventing the algorithm from becoming stuck in local optima.

The sand cat swarm algorithm has a simple structure with few parameters and a low complexity, preventing the algorithm from entering search stagnation by introducing tracking angles; the prey position is not affected by the reduction in the quality of the population. Each member of the population can move independently within different circles, which enables it to obtain better convergence accuracy, though the following problems still occur:

(1) The overall performance of population-based metaheuristic algorithms is significantly affected by the initialization phase. Poor initialization may lead to inefficient exploration of the algorithm, causing it to obey local solutions. Improvement strategies need to be introduced to further strengthen the transition between the algorithm exploration phase and the development phase, and to set a more reasonable sensitivity reduction strategy.

(2) The poor quality of randomly generated populations and a lack of population diversity.

#### 4.3.2. An Improved Mathematical Model for the Optimization of Sand Cat Colonies

For the population intelligence optimization algorithm, ways for achieving balanced switching between global exploration and local exploitation are key to the optimization of the algorithm. In the early iteration of the algorithm, a stronger global exploration ability is needed to maintain the diversity of the population distribution, while in the later iteration, a better local mining ability is needed to ensure the algorithm’s fine mining on the local scale and to accelerate the convergence speed of the algorithm.

In the sand cat swarm algorithm, R plays an important role in the switch between global exploration and local exploitation, $R\in (-r_{G},r_{G})$. During the iteration process, $r_{G}$ decreases linearly in a single cycle, which is inconsistent with the natural law that sand cat populations need multiple rounds of collaboratively rounding up prey, and this also leads to the linear transformation of the fluctuation range of R. For this reason, this paper introduces a nonlinear period adjustment strategy based on logarithmic functions to describe the predation process of sand cat populations.

(28) $$r_{G}=s_{M}-s_{M}\times ln[1+\frac{{iter}_{c}}{{iter}_{max}}{\left(e-1\right)}^{3}]$$

The modified adaptive factor decays slower in early iterations and faster in later iterations. So, the nonlinear cycle adjustment strategy allows the sand cat algorithm to perform a fuller global exploration in the early iterations to maximize population diversity and converge faster in the later iterations. A more balanced and stable switch between global exploration in early iterations and a local development in later iterations has been achieved, further improving the optimization accuracy and convergence speed of the algorithm.

The Opposition-Based Learning (OBL) algorithm improves the performance of population initialization by introducing the inverse element. The basic idea of OBL is to jointly explore any direction and its mirror image while seeking the unknown global optimum, which greatly improves the probability of an individual approaching the optimal solution.

The traditional inverse elements are selected using the method of symmetry about the mean of the upper and lower bounds, but their selection is not flexible enough to quickly and accurately capture the prey. In this paper, we propose a dynamic adjustment method for the search radius based on quasi-inverse learning. In the stage of searching for prey, sand cats need to carry out a large-scale rough search to obtain the approximate location of prey. In the initial stage, individual sand cats should be made to have a large search step. In the stage of launching an attack on the prey, we need to carry out a small-scale detailed search, and in the later stage, the search step should be reduced.

(29) $$x_{b}\left(t+1\right)=\left\{\begin{array}{c} \frac{lb+ub}{2}+\left(\frac{lb+ub}{2}-x\left(t\right)\right)\cdot rand\left(0,1\right),\left|R\right|>1\\ \frac{lb+ub}{2}-\left(\frac{lb+ub}{2}-x\left(t\right)\right)\cdot rand\left(0,1\right),\left|R\right|\leq 1 \end{array}\right.$$

where $x\left(t\right)\in [lb,ub]$ and [lb,ub] is the range of values of sand cat individuals at the current moment t.

### 4.4. DMOCF-Based Neural Network Model

**Figure 5:**
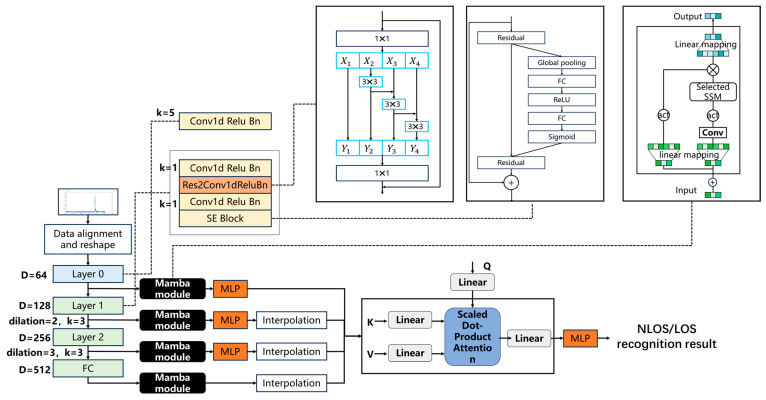
DMOCF network structure diagram.

The DMOCF network model is shown in Figure 5. The CIR peaks of 5G signals are relatively high, and direct and multipath information exists only in very few calendar elements, while the rest of the epoch elements are mostly noisy information. In this regard, we uniformly segment the data at the input end by complementing the zeros and reshaping the data, so that the original unevenly distributed long-sequence data are processed into the splicing of short sequences of data with multiple characterizations of the bottom noise information and few characterizations of the direct and multipath information, which constitutes a two-dimensional data matrix with the same size as the original data. The two-dimensional data matrix of the same size is taken as the original data. The rows of the matrix containing direct and multipath information are contrasted with the other noisy rows, which enables the network to learn the features of the arriving signals efficiently.

Conv1d denotes one-dimensional convolution, which applies a convolution kernel to extract features. Relu is a nonlinear activation function. BN denotes batch normalization, which is used to accelerate the training process of the neural network and improve the stability of the model. The mathematical expression of stacking these three functions together as a basic unit is as follows:

(30) $$y=\frac{\max\left(0,x*w\right)-\mu }{\sqrt{\sigma^{2}+\epsilon }}\cdot \gamma +\beta$$

where x is the input matrix, w is its convolution kernel, ∗ is the convolution operation, max is the maxima-taking operation, μ and $\sigma^{2}$ are the mean and variance of the data that has been convolved with a nonlinear activation function, ϵ is a bias with a very small value preventing the denominator from being zero, γ is the scaling parameter, and β is the translation parameter.

In the comprehensive feature dynamic multilayer hierarchical optimization network proposed in this paper, the above basic unit is used as layer 0. Layers 1 and 2 are added on top of this with the res2 function and the SE block, which implements the learning process of expectation and input discrepancy and enables the input to be passed directly to the subsequent layers by bypassing one or more of the layers. This design resolves the gradient vanishing problem, improves the convergence efficiency of the network, and enables the deeper training of the network.

The Res2 module uses a 3×3 group convolutional layer instead of a 3×3 convolutional layer in the Res module. After the 1×1 convolution of the input sequence, the features are evenly divided into s subsets according to their channel. Except for the first subset, every other feature subset needs to be processed through a 3×3 convolutional layer. Due to the connection operation between subsets, each 3×3 convolutional layer receives information from all its previous feature subsets, resulting in a larger receptive field. The output of the Res2 module includes receptive fields of various sizes, scales, and quantities, as well as their combinations. This grouping and merging strategy enables convolutional layers to express multi-scale features at a finer granularity level, enabling the more effective processing of feature map information. The parameter s is used to control the scale dimension. A larger s can provide more receptive fields of different sizes, but at the same time, it increases computational complexity and memory consumption. In this paper, s=4.

The SE module models the relationship between channels by introducing a squeeze operation and an excitation operation. In the squeezing phase, it compresses the output feature map of the convolutional layer into a feature vector through a global average pooling operation.

(31) $$z=\frac{1}{T}\sum_{t}^{T}h_{t}$$

where $h_{t}$ represents the activations of the last frame layer at time step t. We concatenate the local input $h_{t}$ with the global non-weighted mean and standard deviation of $h_{t}$ across the time domain.

In the excitation phase, a weight vector for a channel is learnt to be generated by using a fully connected layer and a nonlinear activation function.

(32) $$s=\sigma (W_{2}f\left(W_{1}z+b_{1}\right)+b_{2})$$

(33) $${\hat{h}}_{c}=s_{c}h_{c}$$

where σ(·) denotes the sigmoid function that avoids jumps in the output values and provides stability to the gradient computation, and f(·) is a nonlinear operation. $W_{1}\in R^{R\times C}$, $W_{2}\in R^{R\times C}$, $b_{1}\in R^{R\times 1}$, and $b_{2}\in R^{R\times 1}$ denote that the self-attention information is mapped into a smaller R-dimensional space representation that is represented in all C channels and is shared to reduce the risk of parameter computation and overfitting. The resultant vector s contains weights $s_{c}$ between 0 and 1. The weight vector is applied to each channel on the original feature map to weight the features of the different channels. The SE module is able to adaptively learn the importance of each channel and adjust the channel contributions in the feature map weighted according to the needs of the task. This attention mechanism helps the network to better focus on the important feature channels, thus improving the model performance.

In the multilayer network structure based on TDNN, each layer has a strong abstraction and characterization of features and also expresses the relative relationship of data sequences in time. On this basis, we process the TDNN in feature layers by adding a mamba module in each layer to extract high-resolution shallow features and low-resolution fine features, adding position coding in its SSM for the better abstraction of spatial features, both direct and multipath. Each layer obtains different numbers and dimensions of hierarchical feature information. MLP is used in each of the three feature layers with a low number of features to facilitate their dimensional expansion. Interpolation operations were performed on three low dimensional features to ensure consistency in the number and dimension of features at each level. After using the model with hierarchical features, the bottom layer features can be shared and used by multiple upper layer features, the network can learn more abstract and meaningful feature representations, and the feature reuse mechanism can improve the efficiency and generalization ability of the model.

After performing hierarchical feature extraction, we use the self-attention module as a decoder. There are three important input matrices in the self-attention mechanism, the query matrix Q (query), the key matrix K (key), and the value matrix V (value). All three matrices are obtained from the input sequence by different linear transformations, and the product of the query matrix Q and the key matrix K undergoes a softmax function to obtain a probability distribution that is the same as the length of the input sequence, which indicates the importance of each element for the query matrix Q. Multiplying this probability distribution by the value matrix V yields the self-attention vector, which represents the result of weighted average of the values of each element.

(34) $$z=softmax(\frac{Q\times K^{T}}{\sqrt{d}})\cdot V$$

In this paper, a modified sand cat search model is used to search the network parameters so that the loss function is minimized in order to achieve the adaptive tuning of hidden features and the efficient learning of the model.

## 5. Experiments and Results

### 5.1. Experimental Equipment and Signal Processing Flow

**Figure 6:**
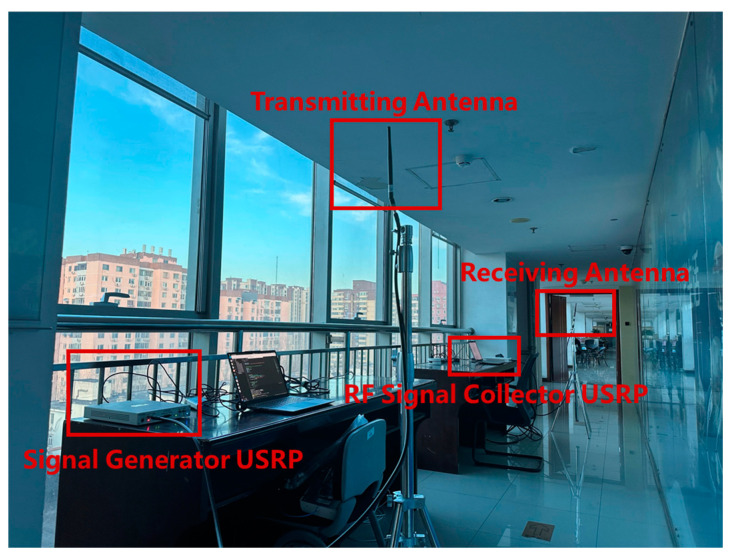
Experimental scene and hardware equipment layout.

**Figure 7:**
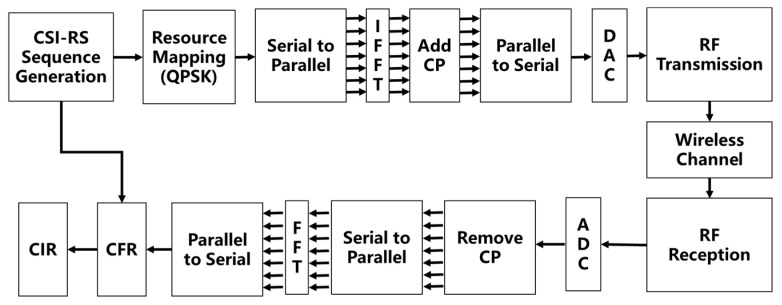
Fifth-generation CIR signal processing flowchart.

In order to evaluate the performance of the algorithm in this paper, a hardware test platform is used to generate the training dataset and the test dataset. USRP is used as the signal transceiver; the hardware platform and the signal processing flow are shown in Figure 6 and Figure 7. The 5G CSI-RS is used as the reference signal for channel estimation, and the CSI-RS is simulated according to the sequence generation rules in 3GPP 38.211, mapped in the time-frequency resource grid using QPSK. After serial-parallel transformation, the IFFT transformation is performed, the obtained time-domain sequence is added with cyclic prefixes, and then, the parallel-serial transformation is performed, broadcasted using USRP X310. The receiver also uses USRP X310 for RF reception, removes the cyclic prefix through the analog-to-digital converter, and then obtains the frequency domain information of the received sequence through the FFT operation. As CSI-RS is used as a frequency-guided signal, its information is transparent to both the receiver and the transmitter, so we can obtain the CFR of the 5G signal by dividing the received frequency domain signal with the local frequency domain signal, and then obtain the CIR data through the IFFT. The CIR data are obtained by IFFT.

### 5.2. Experimental Scenarios and Data Acquisition

**Figure 8:**
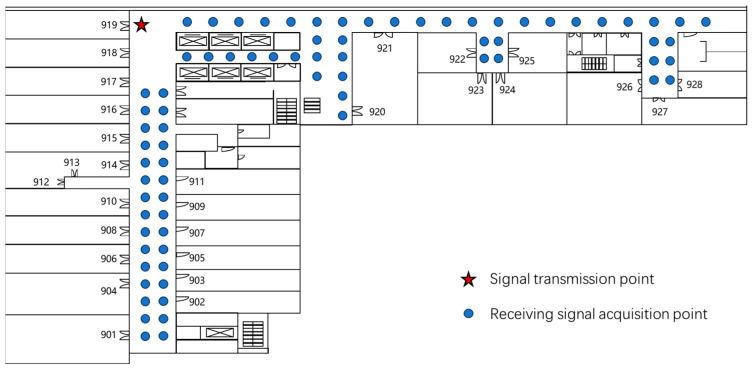
Schematic diagram of data collection points.

In this paper, 5G CIR information was collected in the ninth floor hallway of the research building of the Beijing University of Posts and Telecommunications. The collection point information of the signal source and terminal is shown in Figure 8. A total of 75 points were used to collect the 5G CIR information, among which were 25 NLOS points and 50 LOS points. We conducted measurements 40 times at each point. In order to make the number of the visual distances of the training set and the test set the same, measurements were conducted 30 times at each point in the case of the LOS, and in the NLOS case, 10 times. During NLOS measurement, iron plates were used to artificially block the direct path of wireless signals in the LOS route, from the signal source to the terminal. Before training the model, the 5G CIR dataset was divided into a training set and a test set. The training set used 2000 sets of data and the test set used 1000 sets of data. The proposed LOS/NLOS recognition algorithm was first trained using the training dataset, and then, the algorithm proposed in this paper was validated on the test set.

In order to compare the performance of the LOS/NLOS recognition algorithm proposed in this paper, a CNN-based classifier [36], a FCN-based classifier [37], a LSTM-based classifier [38], a CNN-LSTM-based classifier [38], and a LSTM-FCN-based classifier [39] were also trained. All the deep learning algorithms proposed in the experiments were performed using the open source pytorch framework.

In the network proposed in this paper, Adaptive Moment Estimation (Adam) was used as an optimizer for gradient back propagation. Adam is capable of providing the adaptive learning rate for each parameter, which helps to deal with the situation where the gradient changes by different magnitudes for different parameters. It also introduces the concept of momentum, which is used to update the parameters by calculating the exponentially decaying average of the gradient and helps accelerate convergence and jump out of local optima. In this paper, we used an improved sand cat search algorithm for the efficient search of the initial learning rate.

### 5.3. Experimental Evaluation Indicators

LOS/NLOS recognition can be viewed as a binary classification problem, and therefore, the classification results in the following four cases.

(1) True positive (TP): the true LOS CIR data are classified as LOS CIR data;

(2) True negative (TN): the true NLOS CIR data are classified as NLOS CIR data;

(3) False positive (FP): the true NLOS CIR data are classified as LOS CIR data;

(4) False negative (FN): the true LOS CIR data are classified as NLOS CIR data.

In the experiments, performance metrics such as accuracy (ACC), precision (PPV), recall, specificity, and F1-Score were used to evaluate the performance of the classification algorithm. These elements were calculated as follows:

ACC:

(35) $$ACC=\frac{TP+TN}{TP+FN+TN+FP}$$

PPV:

(36) $$PPV=\frac{TP}{TP+FP}$$

Specificity:

(37) $$Specificity=\frac{TN}{TN+FP}$$

Recall:

(38) $$Recall=\frac{TP}{TP+FN}$$

F1-Score:

(39) $$F1\text{-}Score=\frac{2TP}{2TP+FP+FN}$$

### 5.4. Experimentation and Analysis

**Figure 9:**
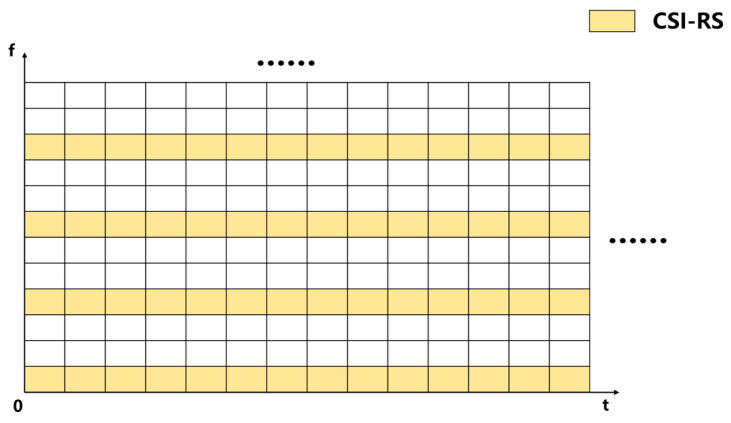
Schematic diagram of 5G CSI-RS time–frequency resource allocation.

In the experiment, the carrier frequency of the 5G signal was 3 GHz; the subcarrier spacing was 60 kHz, occupying 104 resource blocks; the number of samples per symbol was 2048; the 5G CSI-RS adopted non-zero power, occupying 14 symbols per time slot; and the frequency density was 3. The resource configuration schematic is shown in Figure 9.

In this paper, the algorithms were evaluated on a server with an Intel Core i9-11900K CPU, a NVIDIA RTX 3090 graphics card, and 24 Gb RAM.

Due to the relatively simple nature of CIR data and the lack of high dimensionality, the focus of the task was to identify the patterns of signal changes. The current neural network is constantly deepening and becoming more complex, making it prone to overfitting in current tasks. In the experiment, we used commonly used data processing methods in the field of computer vision to increase data diversity, including standardization and data augmentation. The data augmentation methods mainly include adding Gaussian noise, random scaling, and random jitter to enhance data diversity as much as possible to cope with network complexity. On the other hand, some regularization methods can be used to alleviate model overfitting, such as randomly inactivating some neurons through dropout in some intermediate layers. By coordinating complex networks and data, we can address a wider range of data and improve the generalization of the model.

In the experiment, we found that parameters such as batch size and the decay of the learning rate did not affect the training process. Instead, the setting of the learning rate was found to be more important. During the training process, if the learning rate is improperly set, there may be network gradient explosion. Through experimental analysis, setting the network learning rate at around 10^−5^ orders of magnitude can ensure the normal convergence of the network. At the same time, since we segment one-dimensional signals to increase their dimensionality, the positional relationship between different segments is unknown, so positional encoding is necessary.

We conducted ablation experiments to validate the functionality of each module in the network, as shown in Table 1. In the data preprocessing stage, we segmented a long data sequence of 1×2048 into multiple short data sequences of 32×64 by zero padding and reshaped the received data, encoding the position of each segment of data in each level. By incorporating positional encoding, the model can not only learn the content information of the data but also the positional information of the data, thus, better capturing the dependency relationships and patterns in the sequence. It can be seen that adding position encoding can effectively improve LOS/NLOS recognition accuracy while increasing a small number of network parameters and computational consumption. We can see that the inclusion of the mamba module with positional coding can effectively improve the accuracy of NLOS recognition.

**Figure 10:**
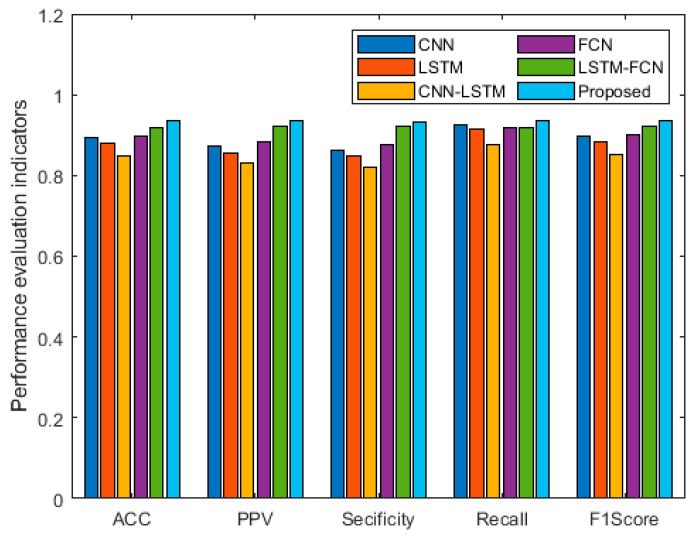
Comparison chart of algorithm performance.

After completing the training, 1000 sets of data were used as the test set, and it can be seen from Table 2 and Figure 10 that the method proposed in this paper correctly recognized 476 line-of-sight CIR data and 459 non-line-of-sight CIR data, with a classification accuracy of 93.5%, which is better than the other comparable algorithms and demonstrates a better NLOS/LOS recognition capability.

We conducted comparative experiments on the complexity of the algorithms, as shown in Table 3. LSTM-based networks have more network parameters and higher computational costs due to their internal gating mechanism and memory units. CNN-based networks have smaller network parameters and computational costs, but due to their inability to perform global modeling, their recognition accuracy is limited. The method proposed in this article has good computational consumption and better recognition accuracy.

## 6. Conclusions

In this paper, we propose a LOS/NLOS recognition method based on DMOCF, which achieves hierarchical feature processing by reshaping the CIR data structure to enhance the contrast between useful and noisy feature learning, in addition to incorporating the res2 module and the SE block in the TDNN to improve the model’s ability to understand the detailed features in the signal in depth. A mamba module with position coding is added to each layer to extract local feature information and global feature information. An improved sand cat search algorithm is proposed for the efficient search of network parameters, which optimizes the predation process of sand cat populations by introducing a nonlinear period adjustment strategy based on the logarithmic function. This also proposes a dynamic adjustment method for the search radius based on quasi-inverse learning, which performs a large-scale coarse search in the initial stage and a small-scale fine search in the prey attack stage, improving search efficiency and accuracy. Overall, the DMOCF algorithm proposed in this article outperforms other methods in LOS/NLOS recognition and has better performance.

Although the method proposed in this article can achieve good LOS/NLOS recognition performance, it requires the collection of prior datasets, resulting in a heavy workload. Ways to expand the data by collecting small sample data are the main research direction for future research to achieve more efficient path state recognition.

## Figures and Tables

**Table 1:** Ablation experiment.

	Network Parameters	Computational Cost	ACC
**TDNN + res2 + SE**	**4.05 M**	**0.21 G**	**81.6%**
**TDNN + res2 + SE + mamba**	**5.64 M**	**0.31 G**	**85.2%**
**TDNN + res2 + SE + mamba + Position encoding**	**5.66 M**	**0.32 G**	**89.1%**
**TDNN + res2 + SE + mamba + Position coding + attention**	**15.09 M**	**0.91** **7 G**	**93.5%**

**Table 2:** Comparison table of algorithms and traditional algorithms used in this article.

Algorithm	TP	FN	FT	TN
**CNN**	**463**	**37**	**68**	**432**
**LSTM**	**456**	**43**	**76**	**425**
**CNN-LSTM**	**438**	**61**	**90**	**411**
**FCN**	**465**	**41**	**61**	**433**
**LSTM-FCN**	**468**	**42**	**39**	**451**
**Proposed**	**476**	**32**	**33**	**459**

**Table 3:** Comparison table of computational complexity.

	Network Parameters	Computational Cost	ACC
**CNN**	**4.718 M**	**1.204 G**	**89.5%**
**LSTM**	**5.242 M**	**9.904 G**	**88.1%**
**CNN-LSTM**	**5.287 M**	**9.992 G**	**84.9%**
**FCN**	**2.098 M**	**4.293 G**	**89.8%**
**LSTM-FCN**	**38.352 M**	**10.327 G**	**91.9%**
**Proposed**	**15.09 M**	**0.91** **7 G**	**93.5%**

## Data Availability

The original contributions presented in the study are included in the article, further inquiries can be directed to the corresponding authors.
